# Gender Differences of Brain Glucose Metabolic Networks Revealed by FDG-PET: Evidence from a Large Cohort of 400 Young Adults

**DOI:** 10.1371/journal.pone.0083821

**Published:** 2013-12-17

**Authors:** Yuxiao Hu, Qiang Xu, Kai Li, Hong Zhu, Rongfeng Qi, Zhiqiang Zhang, Guangming Lu

**Affiliations:** 1 Department of Nuclear Medicine, Jinling Hospital, Clinical school of Medical College, Nanjing University, Nanjing, China; 2 Department of Medical Imaging, Jinling Hospital, Clinical school of Medical College, Nanjing University, Nanjing, China; 3 Department of Pharmacology, Soochow University, Suzhou, China; University G. D'Annunzio, Italy

## Abstract

**Background:**

Gender differences of the human brain are an important issue in neuroscience research. In recent years, an increasing amount of evidence has been gathered from noninvasive neuroimaging studies supporting a sexual dimorphism of the human brain. However, there is a lack of imaging studies on gender differences of brain metabolic networks based on a large population sample.

**Materials and Methods:**

FDG PET data of 400 right-handed, healthy subjects, including 200 females (age: 25∼45 years, mean age±SD: 40.9±3.9 years) and 200 age-matched males were obtained and analyzed in the present study. We first investigated the regional differences of brain glucose metabolism between genders using a voxel-based two-sample *t*-test analysis. Subsequently, we investigated the gender differences of the metabolic networks. Sixteen metabolic covariance networks using seed-based correlation were analyzed. Seven regions showing significant regional metabolic differences between genders, and nine regions conventionally used in the resting-state network studies were selected as regions-of-interest. Permutation tests were used for comparing within- and between-network connectivity between genders.

**Results:**

Compared with the males, females showed higher metabolism in the posterior part and lower metabolism in the anterior part of the brain. Moreover, there were widely distributed patterns of the metabolic networks in the human brain. In addition, significant gender differences within and between brain glucose metabolic networks were revealed in the present study.

**Conclusion:**

This study provides solid data that reveal gender differences in regional brain glucose metabolism and brain glucose metabolic networks. These observations might contribute to the better understanding of the gender differences in human brain functions, and suggest that gender should be included as a covariate when designing experiments and explaining results of brain glucose metabolic networks in the control and experimental individuals or patients.

## Introduction

Gender differences of brain are issues being of continually common interest in neuroscience. In recent years, rapid progress of multi-modality neuroimaging techniques facilitates researches on gender differences of human brain. Structural and functional imaging studies have revealed widely distributed regional differences between genders, both in the cortex and white matter [1], [2], [3], [4], [5], [6]. These findings provide evidences for understanding the neural mechanism of gender differences involved in the cognitive [2], [7], [8], [9], behavioral [10], [11], [12], developmental [3], [13], [14] and disease [15] processes. Moreover, network imaging using different analytic approaches have depicted the gender difference of connectivity properties. Lv et al. [16] had identified gender differences in covariance network of brain morphometry, suggesting advantages of perceptive and high-level cognitive processes in females. Task-based and resting-state Blood oxygen level dependent (BOLD) fMRI also revealed significant gender differences in functional connectivity (FC) [17], [18], [19], [20], [21], [22], which further demonstrated sexual dimorphism in physiological [17], [19], [20], [21] and pathological [18], [22] states.

Functional MRI data was most commonly used to investigate the gender differences of human brain. The most common method of functional MRI is BOLD contrast, a technique which was developed in the 1990s after the BOLD effect was first described [23]. This effect reflects the dependence of T2 weighted contrasts on the amount of blood deoxygenation, which is a consequence of different magnetic properties of oxygenated and deoxygenated hemoglobin. The BOLD contrast, however, only indirectly reflects the neural activity, which is mediated by regional tissue characteristics, oxygen metabolism, cerebral blood flow (CBF) and cerebral blood volume (CBV).

PET is a rather conventional functional imaging modality, and has unique merits in brain investigation by quantitatively measuring the physiological parameters of brain metabolism, blood flow, and receptor density. For example, FDG PET can directly reflect the regional brain glucose metabolism. Moreover, PET has been used to interrogate the brain connectivity from a very early stage by calculating the inter-regional covariance of metabolic values across subjects. The metabolic brain networks constructed by FDG PET reflect the functional connectivity property of the brain [24], and have been widely applied to a cohort of brain diseases [25], [26]. Brain gender difference has been studied using PET in a limited number of literatures. Kawachi et al. [27] have found regional differences in the cortical and sub-cortical structures. Fujimoto et al. [28] have further revealed gender differences in covariance networks in different age stages. Utilizing H_2_^15^O PET, Kilpatrick et al. [29] reported gender-related differences in amygdala network. However, all of these previous studies included relatively small population samples, results from which might affect the analysis of the brain connectivity. Furthermore, Lee et al. [30] have measured the large-scale brain covariance networks using FDG-PET, but the metabolic network based on data of 50 subjects only captured a few network features uncovered by resting-state functional connectivity MRI.

The BOLD signal and FDG PET data reflected the different physiological information, and a recent study found that there was a difference between BOLD functional connectivity and brain metabolic networks [31]. Consequently, although the gender differences of brain networks were found by using functional MRI data in previous studies, these did not demonstrate that there were differences between the genders in brain glucose metabolic networks. In the present study, we re-addressed the brain gender differences in regional and network properties by employing a large cohort of FDG-PET data collected from 400 young healthy subjects. By introducing the newly proposed concepts of large scale brain network in recent findings in imaging research, we presumed that our study provided a more robust imaging presentation for metabolic brain networks, and might contribute to the better understanding of the gender differences in human brain functions.

## Materials and Methods

### 1 Subjects

FDG PET data of 400 right-handed healthy subjects were selected from a data pool consisting of 14,000 cases in the PET center of Jinling Hospital, Clinical school of Medical College, Nanjing University. The PET scans in this paper were not only for the purpose of investigation of gender differences in brain functioning in healthy subjects. In fact, this study is a retrospective study by employing the data out from our clinical PET data pool. I.e., a large proportion of these data scans (346 cases) were performed for health examination for adult disease; and the remaining 54 cases were performed for the following-up of some benign diseases other than CNS disease, including rectal polyp, spherical pneumonia, pulmonary sarcoidosis, etc. All subjects underwent a whole-body PET scan which contained brain scanning and body scanning. In the present study, only brain scanning data was used. The subjects included 200 females (age: 25∼45 years, mean age±SD: 40.9±3.9 years) and 200 age-matched males. All subjects had normal blood pressure and blood glucose, no history of neurological or psychiatric illness (excluded by case history), no alcohol/substance abuse, no cardiovascular disease, and no disease of the digestive system at the time of data acquisition. Written informed consent was obtained from each participant, and this study was approved by the Institutional Review Boards at Jinling Hospital, Clinical school of Medical College, Nanjing University.

### 2 FDG PET imaging acquisition

FDG PET was performed with a Siemens Biograph Sensation 16 PET/CT (Siemens Healthcare, Enlangen, Germany) in three-dimensional mode. Each subject fasted for at least 6 h to keep the blood glucose level at 3.9–6.1 mmol/L. A mean dose of 5.55MBq/Kg (0.15mci/Kg) of FDG was administrated intravenously to each subject. All participants rested for 50–70 min in a quiet room and were scanned while resting with their eyes closed. Brain images were acquired from corona capitis to inferior maxilla plane with an acquisition time of 8–10min. The noncontrast CT scan was performed immediately prior to the PET scan with a multidetector 16 slices spiral CT scanner. The CT data on the combined scanner were used for PET attenuation correction. The FDG PET data were reconstructed with ordered subset expectation maximization (OSEM) iterative algorithm.

### 3 Data preprocessing

The image data preprocessing were conducted using the SPM8 software (http://www.fil.ion.ucl.ac.uk/spm/) based on MATLAB2008 (www.mathworks.com). Since PET data did not contain time-series information, only a single PET image volume was acquired for a subject. The PET image from each subject was spatially normalized to the standard Montreal Neurological Institute (MNI) brain space. During the normalization step, all PET images were resampled at 2×2×2 mm^3^. The spatially normalized PET images were then smoothed by a Gaussian Kernel of 12 mm full-width at half maximum (FWHM). Finally, the PET images were normalized by dividing the whole brain mean signal from the original images.

### 4 Data analysis

**4.1 Comparison of brain glucose metabolism between genders.** Voxel-wised two-sample t-test implemented in SPM 8 was conducted to examine regional metabolic differences between genders. Gender difference was regarded as significant if the corrected *p*<0.05 [height threshold of p<0.05 FDR corrected (T>2.26) and extend threshold of p<0.001 (cluster size >30 voxels)].

**4.2 Construction of Metabolic networks.** Brain glucose metabolic networks were constructed using a seed-based correlation approach [32]. Two sets of regions-of-interest (ROIs) were selected as seed regions. First, seven specific ROIs were post-hoc selected according to the above comparing results between genders Three ROIs were located in the regions that females showing lower metabolism than males, including the left insula (MNI coordinates: –36, –4, –12), the left medial orbitofrontal gyrus(–24, 48, –18) and the left precentral gyrus (–32, –18, 56); two ROIs were located in the cerebellum, including cerebellum-1 (–18, –66, –32) and cerebellum-2 (–18, –58, –56); the remaining two ROIs were located in the regions that females showing higher metabolism than males, including the right thalamus (2, –32, 4) and the left precuneus (–4, –72, 42). These ROIs were defined as spheres centered at the peak coordinates with a radius of 10 mm.

Second, nine ROIs conventionally used for constructing resting-state brain networks were selected in line with the previous studies [33]: the posterior cingulated cortex (PCC) (0, –56, 30), the dorsal medial prefrontal cortex (DMPFC) (0, 54, 30) and the left angular gyrus (AG) (–45, –66, 30), these regions belong to the default mode network (DMN); the right dorsal lateral prefrontal cortex (DLPFC) (42, 45, 26), the left intraparietal lobular (IPL) (–20, –60, 54) and the left frontal eye field (FEF) (–26, 2, 52), these regions belong to the task-positive network (TPN), and correspond to the executive, salience and dorsal attention network, respectively; the left primary somatosensory cortex (SMC) (–50, –24, 46), the left primary visual cortex (VC) (–20, –84, –4) and the left auditory cortex (AC) (–60, –18, 2), belong to the primary sensory networks (SN). The ROIs were also defined as spheres centered at the peak coordinates with a radius of 10 mm. The subject covariance signals were extracted from each ROI, and were then correlated with all brain voxels by using Pearson’s correlation to construct a voxel-wise connectivity map. The methodological approach employed to select ROIs from voxel-wise analysis of maps is routinely used in neuroimaging studies. However, with this approach, the risk to perform a circular analysis (double dipping) is very high. Therefore, the results underwent multiple correction using threshold of *r*>0.21 (corrected *p*<0.05; height threshold of p<0.05, Bonferroni corrected, and extend threshold of *p*<0.001, cluster size > 30 voxels) in the present study. Moreover, region-wise connectivity between ROI pairs was subsequently calculated, and the threshold was assign as *r*>0.21 (corrected *p*<0.05, Bonferroni corrected).

**4.3 Analyze the gender differences within brain metabolic networks.** A nonparametric permutation test with 1000 permutations was used to test the statistical significance of the gender differences within brain networks. The results were thresholded at corrected *p*<0.05 (height threshold of *p*<0.05, Bonferroni corrected, and extend threshold of p<0.001, cluster size > 30 voxels).

**4.4 Analyze the gender differences between brain metabolic networks.** Comparison of region-wise metabolic connectivity between genders were performed using a nonparametric permutation test (1000 permutations) to observe the between-network differences. Gender differences were regarded as significant if the *p*<0.05 (Bonferroni correction).

## Results

### 1 Gender differences in regional brain metabolism

**Figure 1:**
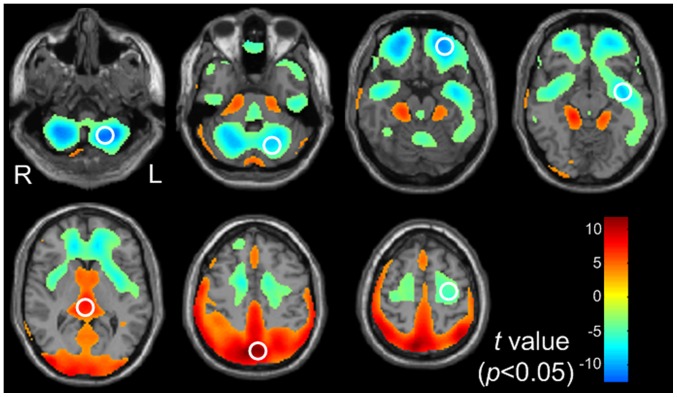
The gender difference maps of regional brain glucose metabolism. Yellow to red areas represent regions where the values of glucose metabolism were higher in females than in males (corrected *p*<0.05) [height threshold of p<0.05 FDR corrected (T>2.26) and extend threshold of p<0.001 (cluster size > 30 voxels)]; The converse is shown as cyan to blue (corrected *p*<0.05) [height threshold of p<0.05 FDR corrected (T>2.26) and extend threshold of p<0.001 (cluster size >30 voxels)]. The white circles indicated the specific ROIs.

The results of the voxel-based analyze (VBA) between the females and the males were shown in Figure 1. Compared with the males, the females had significantly higher glucose metabolism in the posterior brain, including the bilateral posterior parietal lobes, the bilateral occipital lobes, the bilateral thalamus, the bilateral hypothalamus, the bilateral hippocampus and bilateral parahippocampus areas (corrected *p*<0.05)[height threshold of p<0.05 FDR corrected (T>2.26) and extend threshold of p<0.001 (cluster size > 30 voxels)]. While the lower metabolism regions were distributed in the anterior brain, including the bilateral frontal lobes, the bilateral temporal poles, the bilateral anterior parietal cortex, the bilateral insulars, and bilateral cerebellar (corrected *p*<0.05)[height threshold of p<0.05 FDR corrected (T>2.26) and extend threshold of p<0.001 (cluster size > 30 voxels) ].

### 2 Gender differences within brain networks

**Figure 2:**
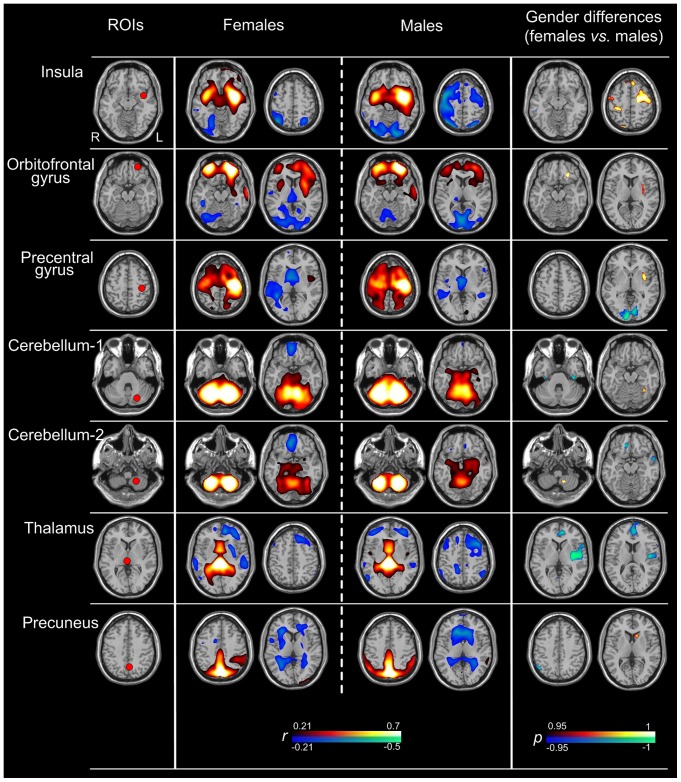
Seven specific brain glucose metabolic network maps of each gender and their differences. The left column displays the locations of the ROIs. The middle two columns show the metabolic networks of both genders. The areas with warm color represented positive correlations with the ROIs, and cold color represented negative correlations (*r*>0.21, corrected *p*<0.05; height threshold of p<0.05, Bonferroni corrected, and extend threshold of *p*<0.001, cluster size >30 voxels). The right column exhibits the main areas of gender differences in brain metabolic networks. The areas with warm color represent increased functional connectivity with each ROI in females than in males; the converse is shown as cold color (corrected *p*<0.05; height threshold of p<0.05, Bonferroni corrected, and extend threshold of *p*<0.001, cluster size > 30 voxels).

**2.1 The gender differences within functional connectivity of 7 specific seeds.** As can be seen in Figure 2, the 7 specific brain glucose metabolic networks of each gender were basically bilateral symmetric. The seed region and contralateral counterpart showed positive correlation with each specific ROI. The positive FC was observed in wide brain areas in the networks correlated with the left insula, the left precentral gyrus, cerebellum-1 and cerebellum-2. Among the 7 specific seeds, 4 seeds were located in the cerebral cortex: including the left insula, left medial orbitofrontal gyrus, precentral gyrus, and left precuneus. In the brain networks correlated with these seeds, negative FC was observed in bilateral cerebellum. Remaining 3 seeds were located in the cerebellum and subcortical area, including cerebellum-1, cerebellum-2 and the right thalamus. In the brain networks correlated with these seeds, negative FC was observed in most cerebral cortex.

The main brain regions of gender differences within the 7 specific seeds functional connectivity were listed in Table 1 (corrected *p*<0.05; height threshold of p<0.05, Bonferroni corrected, and extend threshold of *p*<0.001, cluster size > 30 voxels). All of these 7 brain glucose metabolic networks exhibited significant gender differences.

**Table 1:** The main brain regions of gender differences within seven specific networks.

ROIs	MNI Coordinates	The main brain regions showing increased FC in females	The main brain regions showing increased FC in males
Left insula	–36, –4, –12	Bilateral precentral gyrus, Right postcentral gyrus	Bilateral cerebellum
Left orbitofrontal gyrus	–24, 48, –18	Left middle frontal gyrus, Bilateral postcentral gyrus, Left putamen	None
Left precentral gyrus	–32, –18, 56	Left orbitofrontal gyrus, Left insula	Bilateral lingual gyrus
Cerebellum-1	–18, –66, –32	Left fusiform	Bilateral inferior temporal gyrus
Cerebellum-2	–18, –58, –56	Left cerebellum	Right orbitofrontal gyrus, Left middle Temporal gyrus
Right thalamus	2, –32, 4	Left middle Temporal gyrus	Left insula, Left temporal gyrus, Right cerebellum
Left precuneus	–4, –72, 42	Left caudate	Right angular gyrus

**Figure 3:**
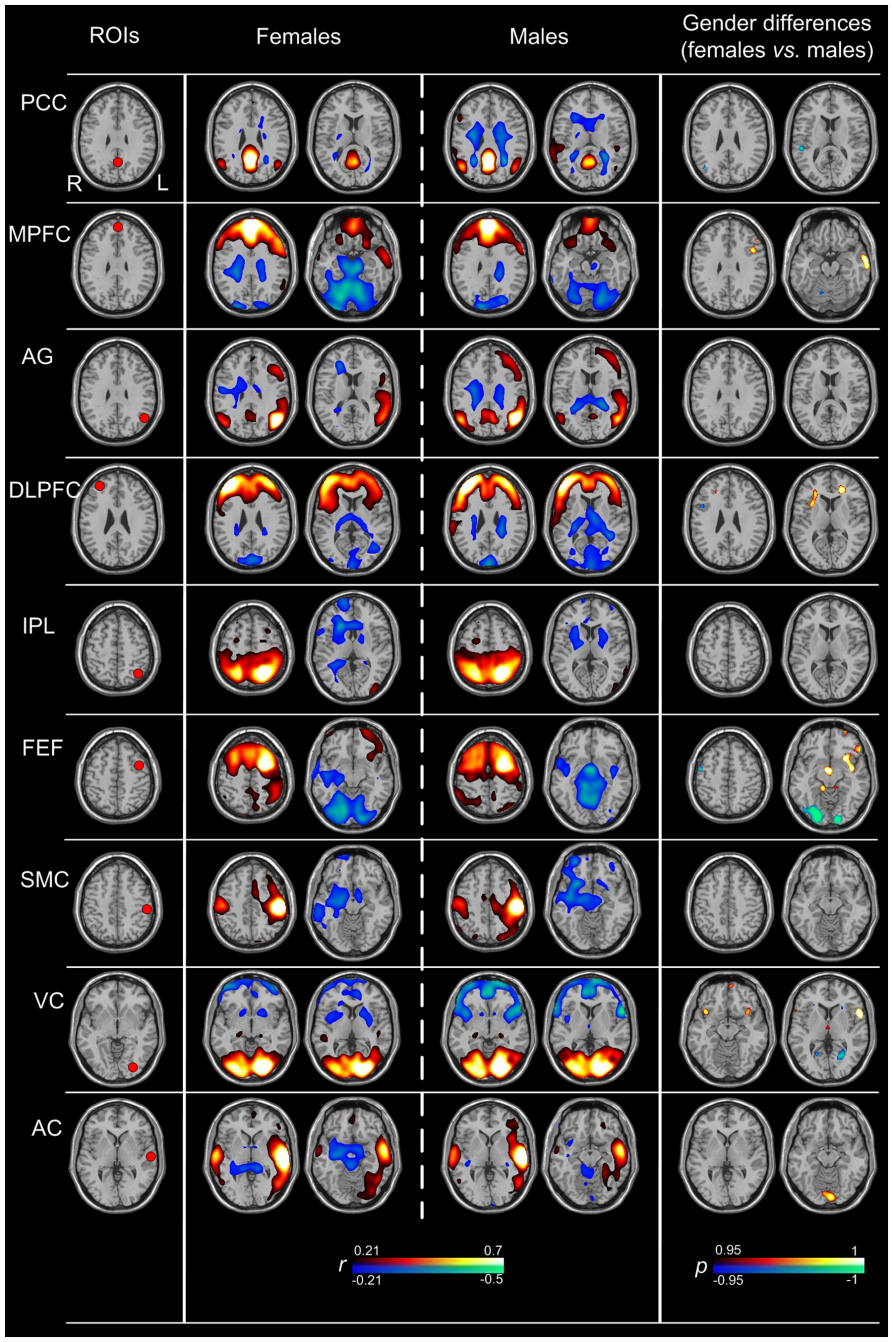
Nine classic brain glucose metabolic network maps of each gender and their differences. The left column displays the locations of the ROIs. The middle two columns show the metabolic networks of both genders. The areas with warm color represented positive correlations with the ROIs, and cold color represented negative correlations (*r*>0.21, corrected *p*<0.05; height threshold of p<0.05, Bonferroni corrected, and extend threshold of *p*<0.001, cluster size > 30 voxels). The right column exhibits the main brain regions of gender differences in brain metabolic networks. The areas with warm color represent increased functional connectivity with each ROI in females than in males; the converse is shown as cold color (corrected *p*<0.05; height threshold of p<0.05, Bonferroni corrected, and extend threshold of *p*<0.001, cluster size > 30 voxels).

**2.2 The gender differences within 9 classic seeds functional networks.** Figure 3 exhibited the metabolic networks correlated with 9 classic seeds of each gender and their differences. Similar with the specific brain networks, 9 classic brain glucose metabolic networks of each gender were basically bilateral symmetric. The seed region and contralateral counterpart showed positive correlation with each classic ROI. Moreover, positive FC was observed in wide areas of cerebral cortex in the networks correlated with PCC, AG, MPFC, IPL and DLPFC. The brain glucose metabolic networks correlated with PCC, AG and MPFC showed typical pattern of DMN, which was similar with that disclosed by fMRI. All 9 classic seeds were located in the cerebral cortex. Negative FC was observed in bilateral cerebellum in most of the classic brain networks except for the network correlated with the VC in both genders.

The main brain regions of gender differences within the 9 classic seeds functional networks are listed in Table 2 (corrected *p*<0.05; height threshold of p<0.05, Bonferroni corrected, and extend threshold of *p*<0.001, cluster size > 30 voxels). Most of the brain glucose metabolic networks showed significant gender differences except for 3 brain networks correlated with AG, IPL, and SMC.

**Table 2:** The main brain regions of gender differences within nine classic networks.

ROIs	MNI Coordinates	The main brain regions showing increased FC in females	The main brain regions showing increased FC in males
PCC	0, –56, 30	None	Right superior temporal gyrus, Bilateral inferior parietal gyrus
MPFC	0, 54, 30	Left middle frontal gyrus, Left precentral gyrus, Left middle temporal	Right cerebellum
AG	–45, –66, 30	None	None
DLPFC	42, 45, 26	Bilateral supplementary motor area, Right putamen, Right superior frontal gyrus	Right inferior frontal gyrus, Left middle temporal gyrus
IPL	–20, –60, 54	None	None
FEF	–26, 2, 52	Left insula, Left orbitofrontal gyrus, Bilateral cerebellum	Bilateral occipital gyrus, Right precentral gyrus
SMC	–50, –24, 46	None	None
VC	–20, –84, –4	Left orbitofrontal gyrus, Bilateral insula, Right inferior frontral gyrus	Bilateral calcarine gyrus
AC	–60, –18, 2	Bilateral Lingual gyrus	Right middle frontal gyrus

### 3 The result of gender differences on correlation between each pair of brain network

**Figure 4:**
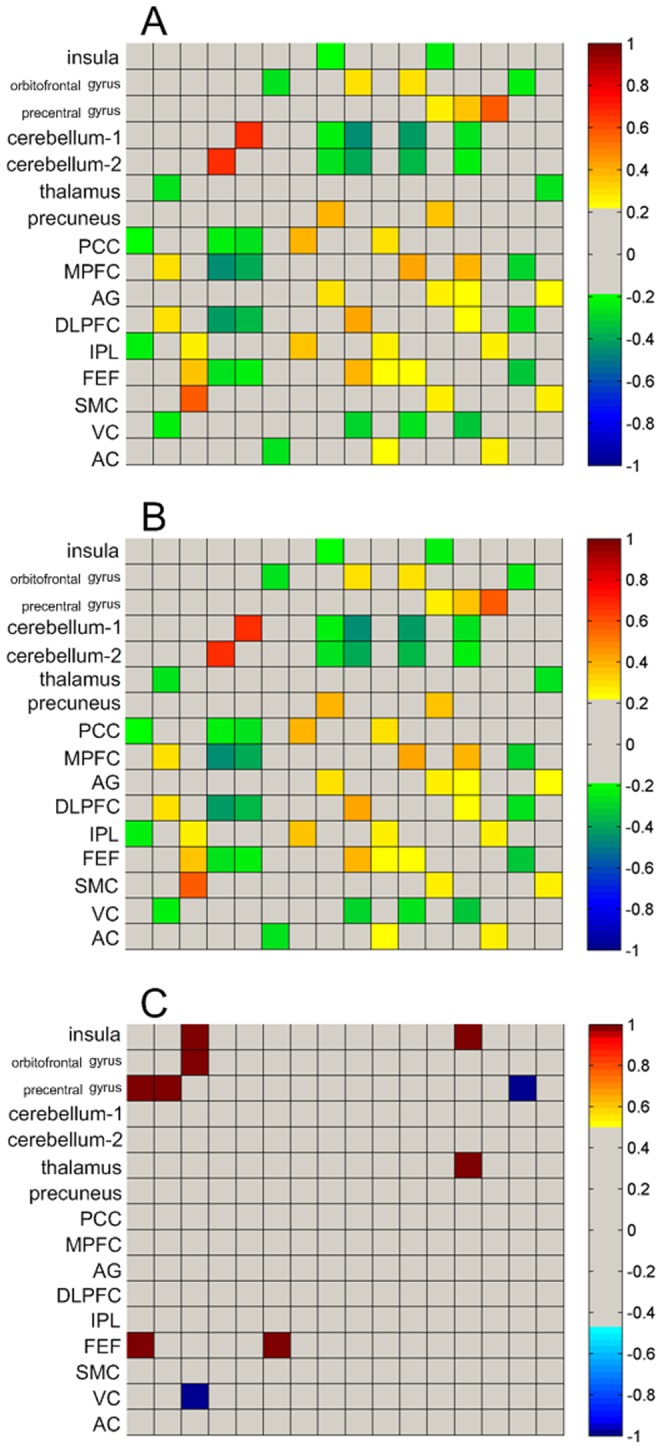
Correlation matrices of 16 ROIs for the females, males and gender differences. A) Inter-subject correlation matrix of females; B) Inter-subject correlation matrix of males; C) corrected gender differences correlation matrix (females-males) (*p*<0.05, Bonferroni correction). In the Figure A and B, warm colors represent positive correlation between a pair of seeds, cold colors represent negative correlation, and gray color represents no correlation. In the Figure C, warm colors represent higher correlations between a pair of seeds in females than in males, cold colors represent lower correlations, and gray color represents that there is not significant differences between genders.

The correlation matrices of 16 ROIs in females, males, and their differences (*p*<0.05, Bonferroni correction) are presented in Figure 4. There are 5 connections exhibited significant gender differences after Bonferroni correction. Among them, 4 connections (brown color): between the left insula and the left precentral gyrus, between the left orbitofrontal gyrys and the left precentral gyrus, between the left insula and the left FEF, and between the right thalamus and the left FEF showed increased correlation coefficient when comparing females to males, and remaining 1 connection (blue color): between the left precentral gyrus and the left VC exhibited decreased correlation coefficient in females than males. Interestingly, all the 5 connections were related with the specific seeds.

## Discussion

Based on a large cohort of FDG PET data from 400 young adults, we investigated the gender difference of brain metabolism in regional and network properties. We observed substantial gender differences in regional brain glucose metabolism. Moreover, widely distributed patterns of the brain glucose metabolic networks in the human brain were observed. Our data exhibited significant gender differences within and between metabolic networks.

VBA is a hypothesis-free, whole-brain, voxel-by-voxel analytical method that attempts to compare neuroimaging data between subjects [34]. Substantial brain regions were involved in gender differences of regional brain glucose metabolism, with some features in the distribution. Firstly, the brain regions of gender differences were symmetric for the left and right hemisphere in most areas. Secondly, the brain regions of gender differences exhibited an interesting distributional pattern of “lower in anterior part” and “higher in posterior part” in the cerebrum, when comparing females to males. In other words, the brain regions of lower glucose uptake values in females than in males were located in the anterior part of the cerebrum, such as frontal cortex, temporal poles, anterior parietal cortex and insular; while the brain regions of higher glucose uptake values in females than in males were located in the posterior part of the cerebrum, such as posterior parietal cortex, occipital cortex, thalamus and hippocampus. Finally, decreased glucose metabolism was found in bilateral cerebellum when comparing the females to the males.

Gender differences in regional brain regions have been reported in many previous neuroimaging studies. In Lv et al.’s [16] study, the greatest gender differences were observed in the bilateral superior frontal gyrus, bilateral precentral gyrus, bilateral postcentral gyrus, and the left superior parietal gyrus, with the females having higher cortical thickness values than the males. Their results are in accordance with the findings shown in other independent studies [35], [36]. But, in our study, the bilateral frontal gyrus and bilateral anterior parietal gyrus exhibited lower glucose metabolism in the females than the males. We suspect that the feature of greater cortical thickness but lower glucose metabolism in some female brain regions may indicate these female brain regions can effectively save energy in resting state. Furthermore, in an early study about gender differences of cerebral glucose metabolism, Kawachi et al. [27] described that the right insula, the middle temporal gyrus and the medial frontal lobe were the regions with metabolisms higher in males than in females, while the hypothalamus exhibited higher metabolism in females than in males. The present study confirmed the results of Kawachi et al.’s study, and observed wider differences in regional brain glucose metabolism between the genders. The wider differences between the genders may be attributed to the larger sample size used the present study.

FDG PET imaging across subject can be used to construct brain networks [25], [26], [31]. The covariance network maybe reflects the specific brain functional connectivity between brain regions. In the previous study of FDG PET, Lee et al. [30] revealed limited inter-subject correlation distributed across the brain by using the seed based approach. They reported three patterns of metabolic connectivity in the human brain: autocorrelation only, correlation with contralatral homologous regions, and correlation with remote areas. In our study, we found positive FC with the seed and the contralateral homologue regions in the 16 brain glucose metabolic networks of both genders, and parts of these metabolic networks showed wide positive correlation with the remote brain regions. The present study obtained the similar results with the Lee et al.’s study. But, some ROIs exhibited wider FC in our study than in Lee et al.’s study, including the left insula, AG, and cerebellum. This may also be due to the larger sample size in the present study. In addition, among the 16 selected seeds, 2 seeds (including cerebellum-1and cerebellum-2) are located in the cerebellum. In the brain metabolic networks correlated with these 2 seeds, negative FC is found in most cerebral cortex. Thirteen seeds are located in the cerebral cortex, negative FC is observed in the bilateral cerebellum in most of the brain networks correlated with these seeds except for the visual network of both genders. These results maybe indicate that there are some specific cerebral regions that anti-correlate with the cerebellum in the resting state.

In recent years, many studies focus on the investigation of the insula function. In the study of Jezzini et al. [37], they studied the insula function in behaving monkeys by using intracortical microstimulation. The results indicated that the monkey’s insula were associated with the sensorimotor function and the mosaic of orofacial motor programs. Another study demonstrated that the anatomical organization of the insula in human is virtually identical to that in the macaque monkey [38]. Therefore, we speculated that the human’s insula were also associated with these 2 functions. In the current study, wide FC was observed between the left insula and the precentral gyrus and the postcentral gyrus in the insula networks of both genders. These results agree with the previous studies. Moreover, the present study found that there were the significant gender differences in some specific brain regions in the insula network. For example, the bilateral precentral gyrus showed increased FC with the left insula in females, which may be related to the stronger sensory function in the females; the bilateral cerebellum exhibited decrease FC with the left insula in females, which may be associated with the weaker balance and motor function in the females.

The cerebellum is one of the regions of the brain that is less understood. Generally, the cerebellum is concerned mainly with motor learning and motor control [39], [40]. The present study observed that the females showed lower regional brain glucose metabolism in bilateral cerebellum than the males, which might be consistent with the weaker motor function of the females. More recently, the cerebellum has been realized to participate in some higher cortical functions, including cognition, behavior, language, and emotion [41], [42], [43]. In the present study, positive FC was observed between cerebellum and bilateral fusiform gyrus in both genders. These results maybe indicate that the cerebellum has involved in the face recognition.

In the present study, we investigated whether there were gender differences within and between the brain metabolic networks. To assure the accuracy of this study, we selected 16 pairs of seeds, including 7 pairs of specific seeds based on our VBA results and 9 pairs of classic seeds based on previous studies [33]. The results showed that significant gender differences were observed in most of the brain glucose metabolic networks except for 3 brain networks correlated with the AG, IPL, and SMC. These indicated that significant gender differences did not just exist within the individual brain glucose metabolic networks, but within most brain glucose metabolic networks. However, the brain regions of gender differences showed in our study were different from those shown in Filippi et al.’s study [22], especially in DMN and SN. These results indicated that the architecture of metabolic networks was inconsistent with the architecture of functional networks [31]. For further study of the gender differences between brain glucose metabolic networks, the present study investigated the gender differences of the correlations between 2 ROIs. After Bonferroni correction, only 4.2% (5/120) connections exhibited significant gender differences. In addition, we found these 5 connections were related to the specific seeds, which might indicate that the seeds showing gender differences in regional brain metabolism were more easily showing gender differences between metabolic networks.

The present study has the largest sample size in studying brain metabolic networks by using FDG PET data. To avoid the affection of normal aging and central nervous system diseases, only young and healthy adults were selected in this study. In the present study, we observed wider brain regions of gender differences than the previous study in regional brain glucose metabolism by using VBA, and first summarized the distributional patterns of these regions. Moreover, we found wider distributed patterns in parts of brain glucose metabolic networks. We first reported that there were significant gender differences within and between brain glucose metabolic networks. With the increasing of FDG PET imaging in the field of neuroscience, the present study may have a practical significance. For example, in the study of brain glucose metabolic networks in some human diseases (e.g. epilepsy, Alzheimer’s disease, Parkinson’s disease, etc.), the proportions of the females and males in both “disease group” and “control group” should be close to 1:1.

There are 2 limitations in the present study. The first limitation is that some of the physiological mechanisms of the gender differences in the brain glucose metabolic networks are still not clear. We speculated that the gender differences of brain glucose metabolic networks directly underlie specific cognitive and behavioral differences between genders, but we lacked direct evidence to support this conclusion. To resolve this problem, more studies regarding the gender effect on brain glucose metabolic networks should be performed by combining with the evaluation of gender-related cognitive performances. The second limitation is that personality characteristics, social learning, and estrogen levels were not taken into account in our study. These factors may affect the regional brain glucose metabolism and the brain glucose metabolic networks [44], [45].

## Conclusion

In summary, this study has accumulated large data supporting the opinion that gender makes a difference in regional brain glucose metabolism and brain glucose metabolic networks. It strongly suggests that gender has a significant influence on the regional neuronal activity in the human brain, perhaps underlying cognitive and behavioral differences between females and males. It should be compulsory to include gender as a covariate when designing experiments and explaining results of brain glucose metabolic networks in healthy and disease subjects. The future works on this topic maybe focus on the following 2 aspects. First, even if the MR does not provide the information of brain glucose metabolism, it can integrate PET data, given its superior spatial and temporal resolution. It is of great significance to comprehensively study the physiological mechanism of the gender differences in the human brain by using multimodal imaging technologies. It can be of help for the design of neuroimaging, neuropsychological and genetic studies. The second direction is to disclose the relation between gender-specific brain glucose metabolic patterns and gender-related differences of various nervous diseases [46], [47].
